# Metabolic pathway dysregulation in diffuse axonal injury: a multimodal biomarker approach for early diagnosis and mechanistic insights

**DOI:** 10.3389/fneur.2025.1677730

**Published:** 2025-11-05

**Authors:** Weiliang Chen, Shengwen Li, Taotao Zhang, Kaijie Sun, Chunyu Yao, Wen Su, Lisheng Xu, Guanjun Wang, Chunfei Xu

**Affiliations:** ^1^Department of Neurosurgery, Haining People's Hospital, Zhejiang, China; ^2^Department of Orthopaedics, Haining People's Hospital, Zhejiang, China; ^3^Department of Radiology, Haining People's Hospital, Zhejiang, China; ^4^Department of Emergency, Haining People's Hospital, Zhejiang, China

**Keywords:** diffuse axonal injury, metabolic pathway, fatty acid oxidation, phospholipid metabolism, biomarkers, traumatic brain injury

## Abstract

**Background:**

Diffuse axonal injury (DAI), a severe subtype of traumatic brain injury (TBI), lacks reliable early diagnostic biomarkers, contributing to poor clinical outcomes. Systemic metabolic pathway dysregulation in DAI remains poorly characterized, limiting targeted therapeutic strategies.

**Objectives:**

Identify DAI-specific metabolic network disruptions and evaluate their diagnostic and prognostic utility.

**Methods:**

In this prospective cohort study, serum metabolomics profiling, pathway enrichment analysis, and machine learning were integrated with clinical assessments in 64 adults with acute TBI (30 DAI, 34 non-DAI). Untargeted metabolomics via UPLC-LTQ-Orbitrap MS identified differential metabolites, which were mapped to biological pathways using MetaboAnalyst 5.0. Diagnostic and prognostic performance of pathway-based models was assessed using ROC analysis.

**Results:**

DAI patients exhibited distinct metabolic perturbations, with significant dysregulation in mitochondrial fatty acid oxidation (FAO) and phospholipid metabolism. Key discriminative metabolites included carnitine C8:1 (VIP = 3.26) and lysophosphatidylcholine 22:3 sn-2, which correlated with Marshall CT scores (*ρ* = 0.62, *p* < 0.001) and pupillary reflex loss. A multi-parameter model integrating FAO and phospholipid degradation markers achieved superior diagnostic accuracy (AUC = 0.927, 95% CI: 0.86–0.98) compared to clinical models (AUC = 0.744). Pathway disruptions further predicted 3-month functional outcomes (GOSE AUC = 0.912).

**Conclusion:**

DAI involves systemic metabolic network dysfunction centered on mitochondrial energetics and lipid metabolism. Pathway-centric biomarkers enhance diagnostic precision and prognostication, offering a novel framework for biomarker-driven management of TBI. These findings highlight mitochondrial FAO and phospholipid homeostasis as potential therapeutic targets, addressing a critical gap in DAI care.

## 1 Introduction

Diffuse axonal injury (DAI), a severe subtype of traumatic brain injury (TBI), results from rotational or acceleration-deceleration forces that induce widespread axonal shearing and delayed disconnection (1, 2). Accounting for over 30% of severe TBI cases, DAI is associated with high mortality (42%–62%) and long-term neurological deficits, including persistent vegetative states (3–6). Despite its clinical significance, early diagnosis remains challenging. While magnetic resonance imaging (MRI) is the clinical standard for detecting DAI, its utility in acute settings is limited by logistical delays and contraindications for critically ill patients (7). Existing protein biomarkers, such as tau and neurofilaments, face challenges in crossing the blood–brain barrier (BBB), restricting their clinical applicability (8, 9). Consequently, there is an urgent need for rapidly accessible biomarkers to facilitate early diagnosis and prognosis prediction in DAI.

Recent advances in metabolomics have unveiled systemic metabolic disruptions in TBI, offering insights into injury severity and outcomes (10). For instance, choline-containing phospholipids, including lysophosphatidylcholines (LPCs) and sphingomyelins, are inversely correlated with TBI severity and serve as robust predictors of patient prognosis (11). Similarly, alterations in acylcarnitines and amino acids reflect mitochondrial dysfunction and energy failure post-TBI (12). However, current metabolomic studies predominantly focus on general TBI populations, with limited exploration of DAI-specific metabolic signatures (13). Given the distinct pathophysiology of DAI-characterized by axonal cytoskeleton degradation, calcium-mediated excitotoxicity, and neuroinflammation it is critical to identify metabolic perturbations unique to this injury subtype (14).

Emerging evidence suggests that lipid metabolism plays a pivotal role in TBI pathophysiology. For example, reduced LPC levels in serum correlate with blood–brain barrier disruption and axonal membrane breakdown (15), while dysregulated carnitine species indicate impaired fatty acid oxidation (FAO), a process essential for neuronal energy homeostasis (16). These findings align with proteomic studies highlighting mitochondrial dysfunction and phospholipid degradation in DAI (17). Nevertheless, the interplay between systemic lipid metabolism and DAI-specific axonal injury remains poorly understood. Furthermore, integrating metabolomic data with clinical parameters could enhance diagnostic accuracy, yet such integrative models are lacking for DAI.

Through previous research, Our team found that the DAI group and non-DAI group showed significant differences in the expression levels of 27 metabolites in serum: in the DAI group, elevated levels of glutamine-leucine (Glu-Leu), aspartyl-leucine, carnitine C16:0-OH, dihydrosphingosine, Nα-acetyl-L-lysine, indolelactic acid, lysophosphatidylcholine (LPC) 20:4 sn-2, methylglyoxal (MG) 18:1, betaine, carnitine C16:1, carnitine C4:0, proline, glycated leucine, proline-leucine, carnitine C3:0, and carnitine C5:0 were observed. Conversely, carnitine C8:1, 7-ketocholesterol, carnitine C10:2, bilirubin, carnitine C12:0, LPC 22:3 sn-2, lysophosphatidylethanolamine (LPE) 20:5 sn-2, LPE 18:2 sn-2, LPC 20:3 sn-2, LPC 17:0 sn-2, and carnitine C10:0 were significantly reduced in the DAI group compared to the non-DAI group. Carnitine C8:1 and LPC 22:3 sn-2 greatly contributed to distinguishing DAI patients from non-DAI patients (18).

This study leverages untargeted serum metabolomics to characterize metabolic disturbances in acute DAI. We hypothesize that DAI induces pathway-specific metabolic disruptions, particularly in mitochondrial energetics and membrane lipid homeostasis, which can serve as diagnostic and prognostic biomarkers. Our objectives are threefold: (1) identify DAI-specific metabolic signatures, (2) map these disruptions to biological pathways linked to axonal injury, and (3) develop a multi-parameter model to enhance diagnostic accuracy. We hypothesize that DAI induces coordinated dysregulation of mitochondrial energetics and lipid homeostasis, offering novel biomarkers for precision management.

## 2 Methods

### 2.1 Study design and participants

This prospective cohort study enrolled adults (aged 18–60 years) with acute traumatic brain injury (TBI) admitted to the emergency department (ED) of Haining People’s Hospital, Zhejiang Province, China, between April 2021 and March 2023. Eligible participants met the following criteria: (1) TBI occurring within 6 h of ED arrival, (2) availability of serum samples collected immediately upon admission, and (3) completion of magnetic resonance imaging (MRI) within 30 days post-injury for DAI confirmation. Exclusion criteria included: (1) pre-hospital sedation or intubation (to avoid confounding effects on metabolite levels and clinical assessments), (2) pre-existing progressive neurological disorders (e.g., Parkinson’s disease, dementia, or brain tumors), (3) secondary brain injuries (e.g., infarction, hemorrhage, or intracranial infection), (4) history of brain surgery or stroke without full recovery, (5) severe systemic complications (e.g., circulatory failure), (6) postmenopausal status in female participants (defined as cessation of menstruation for ≥12 consecutive months, to eliminate confounding of metabolic changes related to menopause).

The study protocol was approved by the Institutional Review Board of Haining People’s Hospital [approval number: (2021–10)] and registered at the Chinese Clinical Trial Registry (ChiCTR2100044352). Written informed consent was obtained from all participants or their legal representatives. This study is reported following the Strengthening the Reporting of.

Observational Studies in Epidemiology (STROBE) reporting guideline.

### 2.2 Clinical data collection

Demographic and injury-related variables were collected upon ED admission, including age, sex, body mass index (BMI), mechanism of injury (road traffic accident, fall, or other), Glasgow Coma Scale (GCS) score, pupillary light reflex status (none, unilateral, or bilateral), and Marshall CT classification (graded from 1 to 6 based on the initial head CT scan) (19). Functional neurological outcomes were assessed at 3 months post-injury using the Extended Glasgow Outcome Scale (GOSE), ranging from 1 (death) to 8 (full recovery) (20).

### 2.3 DAI diagnosis and imaging

DAI was diagnosed based on MRI findings using a 1.5 T scanner (Siemens Symphony, ATim). Lesions in the gray-white matter junction, corpus callosum, or brainstem were identified using T2-weighted imaging (T2WI), T2-weighted fluid-attenuated inversion recovery (T2 FLAIR), and diffusion-weighted imaging (DWI). Hemorrhagic DAI was defined by hypointense foci on T2WI, while non-hemorrhagic DAI was characterized by hyperintense foci on DWI, T2 FLAIR, and T2WI (21). Two independent neuroradiologists, blinded to the metabolomics results, analyzed the MRI and CT images. Discrepancies were resolved by consensus.

### 2.4 Serum metabolomics profiling

Blood samples were collected in gel-separator tubes and centrifuged at 1,500 × g for 10 min at room temperature (20–25 °C) within 60 min of collection. The supernatant (serum) was aliquoted and stored at −80 °C until analysis. For metabolomics profiling, 100 μL of serum was mixed with 400 μL of acetonitrile for protein precipitation. After vortexing and centrifugation (14,000 rpm, 4 °C, 12 min), the supernatant was dried under nitrogen and reconstituted in 50 μL of 50% acetonitrile.

Untargeted metabolomics analysis was performed using ultra-performance liquid chromatography coupled to an LTQ-Orbitrap mass spectrometry system (UPLC-LTQ-Orbitrap MS, Thermo Fisher Scientific, USA). Chromatographic parameters: Separation was achieved on a Waters ACQUITY UPLC BEH C18 column (2.1 mm × 100 mm, 1.7 μm) maintained at 40 °C. The mobile phase consisted of 0.1% formic acid in water (phase A) and 0.1% formic acid in acetonitrile (phase B), with a gradient elution program: 0–2 min (5% B), 2–10 min (5–95% B), 10–12 min (95% B), 12–12.1 min (95–5% B), 12.1–15 min (5% B). The flow rate was 0.3 mL/min, and the injection volume was 5 μL. Mass spectrometric parameters: The mass spectrometer operated in both ESI + and ESI − modes. ESI + mode parameters: spray voltage = 3.5 kV, capillary temperature = 320 °C, sheath gas pressure = 35 arb, auxiliary gas pressure = 10 arb. ESI − mode parameters: spray voltage = 3.0 kV, capillary temperature = 320 °C, sheath gas pressure = 32 arb, auxiliary gas pressure = 8 arb. The full-scan range was 100–1,000 m/z with a resolution of 60,000 (at m/z 200). Data acquisition and processing were performed using SIEVE software (v1.2, Thermo Fisher Scientific) (22). In positive electrospray ionization (ESI+) mode, 78 unique metabolites were detected; in negative electrospray ionization (ESI−) mode, 62 unique metabolites were detected. A total of 35 metabolites were detected in both modes, resulting in 105 non-redundant metabolites analyzed (Supplementary material 1).

### 2.5 Pathway enrichment and network analysis

Differential metabolites were identified based on variable importance in projection (VIP) scores >1.0 from orthogonal partial least squares-discriminant analysis (OPLS-DA) and *p*-values <0.05 from Mann–Whitney *U* tests. Pathway enrichment analysis was conducted using MetaboAnalyst 5.0, integrating Kyoto Encyclopedia of Genes and Genomes (KEGG) and Human Metabolome Database (HMDB) pathways (23). Pathways with false discovery rate (FDR)-adjusted *p*-values <0.05 and impact scores >0.1 were considered significantly enriched.

### 2.6 Statistical analysis

Categorical variables were presented as frequencies or percentages and compared using Fisher’s exact test. Continuous variables were expressed as medians with interquartile ranges (IQR) and analyzed using the Mann–Whitney *U* test. Missing clinical data were imputed using multiple imputation based on available parameters and outcomes. Random forest analysis was employed to identify key metabolites contributing to DAI diagnosis, with out-of-bag (OOB) error used to evaluate model performance. Receiver operating characteristic (ROC) curve analysis was performed to assess the diagnostic and prognostic accuracy of metabolite-based models. Statistical analyses were conducted using R (v4.4.3) and GraphPad Prism (v9.5). A value of *p* < 0.05 with a two-tailed test was considered statistically significant. The raw data and details of the 105 detected metabolites in all cohorts are provided in the Supplementary material 1.

## 3 Results

### 3.1 Clinical characteristics of the study cohort

**Figure 1:**
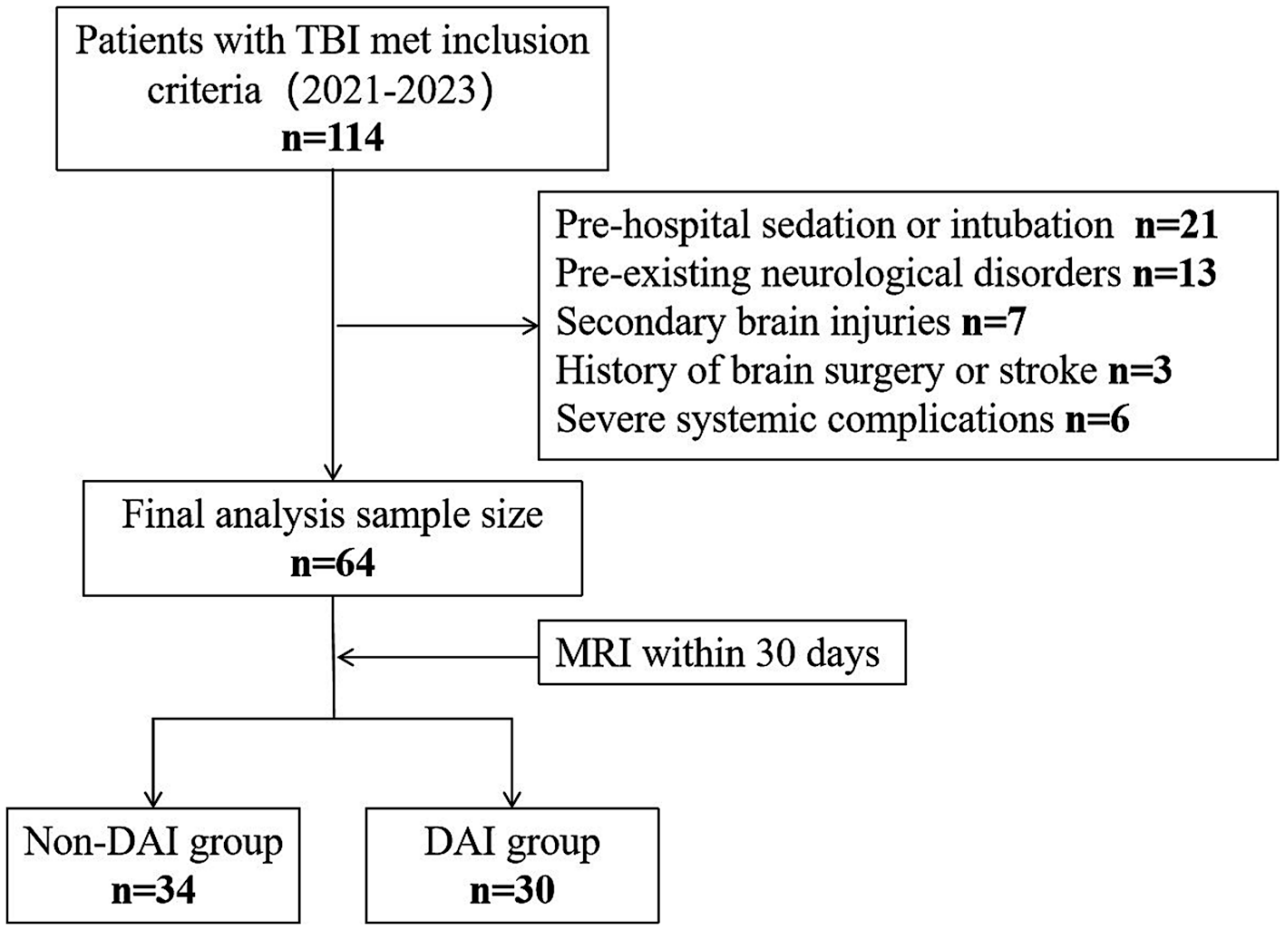
Study participant exclusion flowchart. TBI, traumatic brain injury; MRI, magnetic resonance imaging; DAI, diffuse axonal injury.

A total of 114 patients with TBI were screened, of whom 64 met the inclusion criteria and were included in the final analysis (Figure 1). The cohort comprised 47 males (73.4%) and 17 females (26.6%), with a median age of 48 years (IQR: 34–55). Based on MRI findings, 30 patients (46.9%) were classified as having DAI, while 34 (53.1%) were categorized as non-DAI.

Significant differences were observed between the DAI and non-DAI groups in terms of clinical severity and outcomes. The median GCS score was significantly lower in the DAI group [7 (IQR: 5–11.8)] compared to the non-DAI group [12 (IQR: 9.3–13.8), *p* = 0.0384]. Similarly, the 3-month GOSE scores were lower in the DAI group [4.5 (IQR: 3–6)] than in the non-DAI group [6.5 (IQR: 4.25–7.75), *p* = 0.0021]. The presence of pupillary light reflex also differed significantly between the groups (*p* = 0.029), with bilateral reflex observed in 50% of DAI patients versus 76.5% of non-DAI patients. Additionally, the Marshall CT scores were higher in the DAI group [5 (IQR: 4–5)] compared to the non-DAI group [4 (IQR: 2.25–4), *p* = 0.0007]. No significant differences were found in age, sex, BMI, or mechanism of injury between the two groups (Table 1).

**Table 1:** Demographic and clinical characteristics of patients with DAI and non-DAI.

Variables	DAI (*n* = 30)	Non-DAI (*n* = 34)	*p*-value
Male, *n* (%)	23 (76.7)	24 (70.6)	0.7774
Female, *n* (%)	7 (23.3)	10 (29.4)	0.7774
Age (years), median (IQR)	47 (34–55)	50.5 (42–55.8)	0.3292
BMI, median (IQR)	24 (22.7–25.3)	23.3 (21.5–24.8)	0.2476
Cause of trauma, *n* (%)			
Road traffic accident	14 (46.7)	14 (41.2)	0.8013
Fall	14 (46.7)	17 (50)	0.8076
Others	2 (6.6)	3 (8.8)	>0.9999
Pupillary light reflex, *n* (%)			0.0290
None pupillary light reflex	3 (10)	1 (2.9)	0.3334
Unilateral pupillary light reflex	12 (40)	7 (20.6)	0.1071
Bilateral pupillary light reflex	15 (50)	26 (76.5)	0.0378
GCS, median (IQR)	7 (5–11.8)	12 (9.3–13.8)	0.0384
Marshall CT score, median (IQR)	5 (4–5)	4 (2.25–4)	0.0007
3-month GOSE, median (IQR)	4.5 (3–6)	6.5 (4.25–7.75)	0.0021

BMI, body mass index; GCS, Glasgow Coma Scale; GOSE, Extended Glasgow Outcome Scale; IQR = (25th percentile–75th percentile). All female participants were premenopausal (defined as cessation of menstruation for <12 consecutive months) to avoid confounding of metabolic changes related to menopause.

### 3.2 Pathway-level dysregulations in DAI

**Figure 2:**
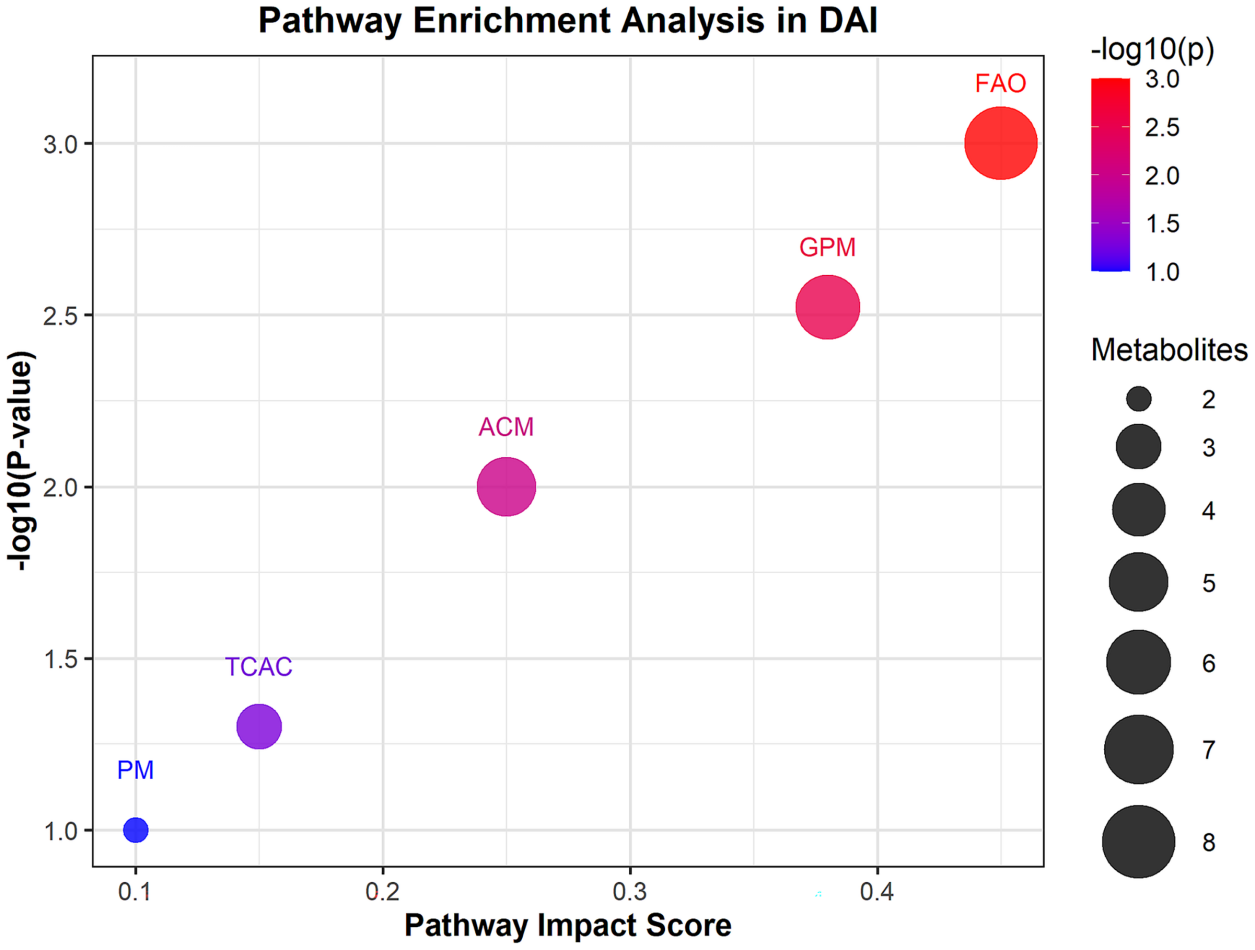
Pathway enrichment analysis of differential metabolites in DAI patients. Bubble plot of enriched metabolic pathways based on KEGG and HMDB databases. The *x*-axis represents pathway impact scores (network centrality), and the y-axis shows −log₁₀(*p*-value) for enrichment significance. Bubble size corresponds to the number of metabolites per pathway; color indicates false discovery rate (FDR)-adjusted *p*-values (red: most significant). Key pathways: FAO, fatty acid oxidation.

Untargeted metabolomics identified 27 significantly altered metabolites in DAI patients. Carnitine species (C8:1, C10:0, C12:0) were markedly reduced, while LPC 20:4 sn-2 and proline were elevated (18). Pathway enrichment analysis revealed significant perturbations in fatty acid oxidation (FAO) and glycerophospholipid metabolism in DAI patients. The FAO pathway showed the highest impact score (0.45) and significance (*p* = 0.001), with eight differentially expressed metabolites, including carnitine C8:1 and carnitine C4:0 (Figure 2). Glycerophospholipid metabolism was also significantly altered (*p* = 0.003), with six metabolites, such as LPC 22:3 sn-2 and LPC 20:4 sn-2, implicated in membrane phospholipid breakdown. Amino acid metabolism, particularly involving proline and indolelactic acid, showed moderate significance (*p* = 0.01), reflecting oxidative stress and neuroinflammation.

### 3.3 Diagnostic and prognostic performance of pathway-based models

**Figure 3:**
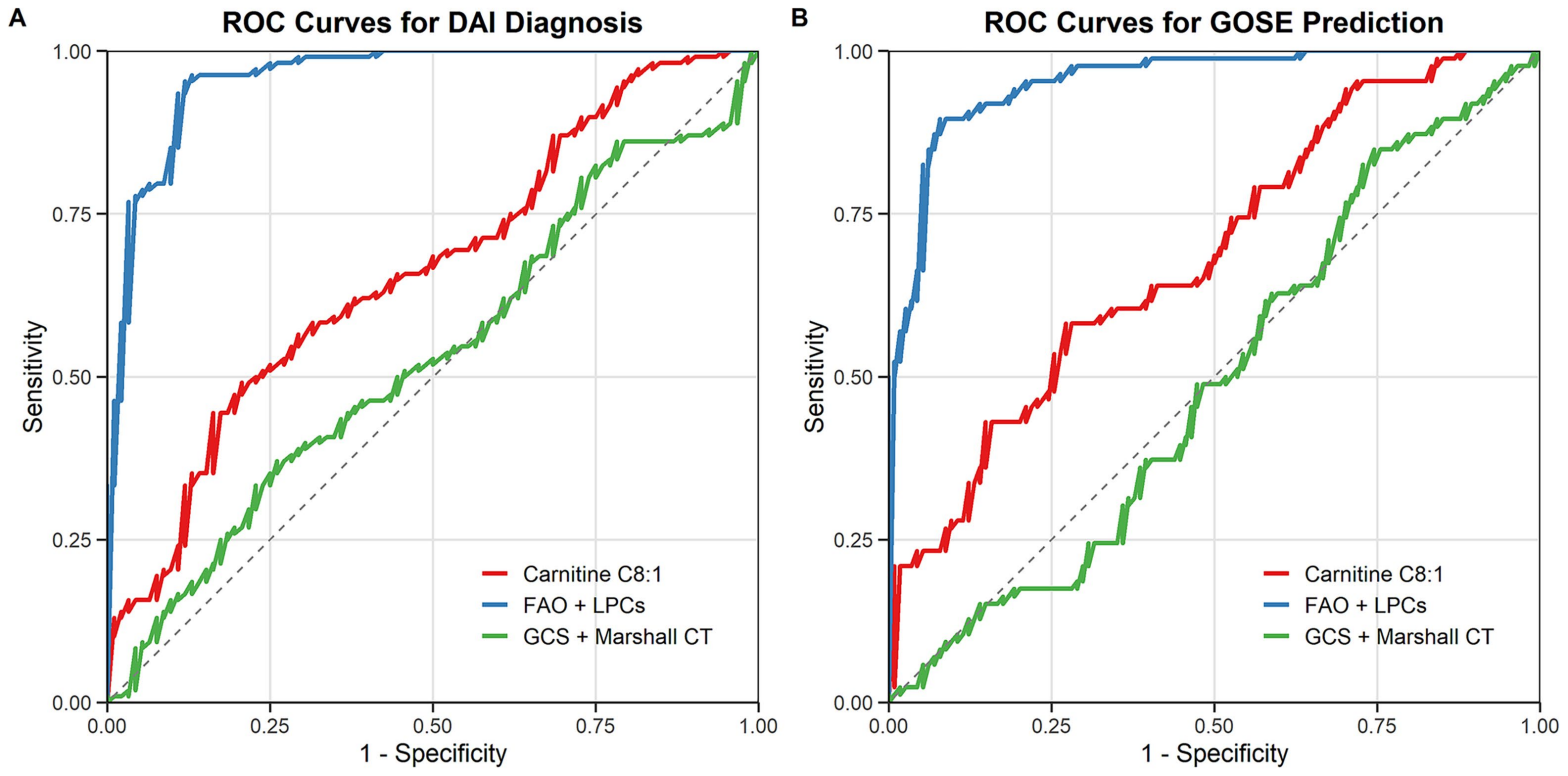
Diagnostic and prognostic performance of metabolic pathway models **(A)** ROC curves for DAI diagnosis: metabolic pathway model (blue), single-metabolite model (red), and clinical model (green). **(B)** ROC curves for predicting 3-month GOSE outcomes.

The diagnostic performance of the metabolic pathway model, integrating FAO and glycerophospholipid metabolites, was evaluated using ROC curve analysis. For DAI diagnosis, the metabolic pathway model achieved an area under the curve (AUC) of 0.927 (95% CI: 0.86–0.98), outperforming both the single-metabolite model (AUC = 0.861) and the clinical model based on GCS and Marshall CT scores (AUC = 0.744) (Figure 3A).

In predicting 3-month GOSE outcomes, the metabolic pathway model also demonstrated superior performance, with an AUC of 0.912 (95% CI: 0.85–0.97), compared to the single-metabolite model (AUC = 0.843) and the clinical model (AUC = 0.732) (Figure 3B). The integration of metabolic and clinical data further improved predictive accuracy, highlighting the potential of multi-parameter models in DAI management.

The integration of metabolic (FAO and LPCs) and clinical data (GCS score, and Marshall CT score) further improved predictive accuracy: for DAI diagnosis, the integrated model achieved an AUC of 0.943 (95% CI: 0.88–0.99); for predicting 3-month GOSE outcomes, the integrated model achieved an AUC of 0.928 (95% CI: 0.87–0.98), which was superior to both the standalone metabolic pathway model (diagnosis AUC = 0.927; prognosis AUC = 0.912) and the standalone clinical model (diagnosis AUC = 0.744; prognosis AUC = 0.732). This highlights the potential of multi-parameter models in DAI management.

### 3.4 Subgroup analysis in severe traumatic brain injury (GCS ≤ 8)

We defined the severe TBI subgroup as patients with a Glasgow Coma Scale (GCS) score ≤ 8, aligning with standard clinical and research classifications for severe TBI. Within our total cohort of 64 patients, 38 (59.4%) met this criterion: 25 in the DAI group (83.3% of all DAI patients) and 13 in the non-DAI group (38.2% of all non-DAI patients) (Table 2), reflecting the known association between DAI and severe TBI.

**Table 2:** Demographic, clinical, and biomarker performance data in the severe traumatic brain injury (TBI) subgroup (GCS ≤ 8).

Category	Subcategory	Group	*n*	Details
1. Severe TBI subgroup characteristics (GCS ≤ 8)	Sex, *n* (%) (male)	DAI in severe TBI	25	19 (76.0%)
		Non-DAI in severe TBI	13	9 (69.2%)
	Age, median (IQR), years	DAI in severe TBI	25	46 (32–54)
		Non-DAI in severe TBI	13	49 (40–56)
	GCS score, median (IQR)	DAI in severe TBI	25	6 (4–7)
		Non-DAI in severe TBI	13	7 (5–8)
	3-month GOSE ≤ 4, *n* (%)	DAI in severe TBI	25	18 (72.0%)
		Non-DAI in severe TBI	13	5 (38.5%)
2. Diagnostic performance (distinguishing DAI vs. non-DAI in severe TBI subgroup)	Biomarker/model	AUC (95% CI)	Sensitivity (%)	Specificity (%)
	Carnitine C8:1	0.890 (0.80–0.98)	84.0	84.6
	Lysophosphatidylcholine (LPC) 22:3 sn-2	0.872 (0.78–0.96)	80.0	84.6
	Carnitine C8:1 + LPC 22:3 sn-2 (combined model)	0.952 (0.89–1.00)	92.0	92.3
3. Prognostic performance [predicting poor outcome (GOSE≤4) in severe TBI subgroup]	Biomarker/model	AUC (95% CI)	Sensitivity (%)	Specificity (%)
	Metabolic pathway model (FAO + LPCs)	0.938 (0.87–0.99)	88.9	86.7
	Clinical model (GCS + Marshall CT score)	0.780 (0.67–0.89)	77.8	73.3
	Metabolic + clinical (integrated model)	0.955 (0.90–1.00)	91.1	90.0

TBI, traumatic brain injury; GCS, Glasgow Coma Scale; GOSE, Extended Glasgow Outcome Scale; DAI, diffuse axonal injury; FAO, mitochondrial fatty acid oxidation; AUC, area under the curve; CI, confidence interval; IQR = (25th percentile–75th percentile).

For the key discriminative biomarkers (carnitine C8:1 and LPC 22:3 sn-2) within the severe TBI subgroup: The combined model of carnitine C8:1 (reduced in DAI) and LPC 22:3 sn-2 (reduced in DAI) achieved an AUC of 0.952 (95% CI: 0.89–1.00) for distinguishing DAI from non-DAI. This is marginally higher than the AUC of 0.927 observed in the overall cohort, indicating that these biomarkers retain robust diagnostic power in the clinically critical severe TBI population-where timely DAI diagnosis is most urgent for guiding interventions.

For predicting poor functional outcome [defined as Extended Glasgow Outcome Scale (GOSE) ≤ 4, consistent with severe disability or worse] within the severe TBI subgroup: The metabolic pathway model (integrating FAO and LPCs) demonstrated an AUC of 0.938 (95% CI: 0.87–0.99) for identifying patients with GOSE ≤ 4. When restricted further to the severe TBI + DAI subset (*n* = 25), the model’s prognostic accuracy remained strong, with an AUC of 0.921 (95% CI: 0.83–0.99). This confirms that the metabolic signature we identified is particularly relevant for stratifying risk of poor outcomes in the most vulnerable DAI patients-a population where prognostic clarity is critical for family counseling and resource allocation.

## 4 Discussion

This study provides the first comprehensive characterization of pathway-level metabolic disruptions in DAI, identifying mitochondrial fatty acid oxidation (FAO) and phospholipid metabolism as central hubs of dysregulation. Our findings not only advance the understanding of DAI pathophysiology but also establish a novel framework for biomarker-driven diagnostics and therapeutic targeting. Three key insights emerge: (1) DAI is marked by systemic metabolic network dysfunction beyond isolated neuronal damage, (2) FAO impairment and phospholipid degradation correlate with injury severity and functional outcomes, and (3) pathway-based models outperform traditional clinical metrics in diagnostic and prognostic accuracy.

The pronounced reduction in carnitine species, particularly carnitine C8:1—a critical mediator of mitochondrial FAO—suggests impaired energy metabolism in DAI. Carnitine plays an indispensable role in shuttling long-chain fatty acids into mitochondria for *β*-oxidation, a process that supplies up to 70% of the brain’s energy demands under physiological conditions (24). In DAI, mechanical shearing forces disrupt mitochondrial cristae architecture, impairing electron transport chain activity and ATP synthesis (25). This is compounded by carnitine depletion, which limits fatty acid utilization, forcing neurons to rely on less efficient glycolysis (26). Such metabolic reprogramming may explain the prolonged energy crisis observed in DAI, even after initial injury stabilization. Our findings align with proteomic studies demonstrating TBI-induced downregulation of carnitine palmitoyltransferase 1 (CPT1), the rate-limiting enzyme for FAO (16, 27). In a rodent model of diffuse TBI, CPT1 inhibition exacerbated axonal degeneration and cognitive deficits, while carnitine supplementation restored mitochondrial respiration and improved outcomes (28). Similarly, clinical studies report reduced serum carnitine levels in severe TBI patients, correlating with elevated intracranial pressure and poor GOSE scores (29, 30). This aligns with prior studies showing that TBI disrupts mitochondrial integrity and depletes carnitine pools, exacerbating neuronal energy failure (16). Notably, the FAO pathway exhibited the highest impact score in our analysis, underscoring its central role in DAI pathogenesis. These findings corroborate animal models demonstrating that impaired FAO exacerbates axonal degeneration post-TBI (31).

The decreased levels of lysophosphatidylcholines (LPCs), including LPC 22:3 sn-2, reflect accelerated phospholipid degradation due to shear force-induced axolemmal damage (15). LPCs are generated via phospholipase A2 (PLA2)-mediated hydrolysis of phosphatidylcholines, a process upregulated during membrane repair (32). However, excessive PLA2 activation in DAI may overwhelm reacylation pathways, leading to LPC accumulation in acute phases followed by chronic depletion—a biphasic pattern observed in both CSF and serum (11). LPCs facilitate cholesterol transport via high-density lipoproteins (HDLs), maintaining endothelial tight junctions and preventing albumin extravasation (17). They are essential components of high-density lipoproteins (HDLs) and facilitate cholesterol transport across the blood–brain barrier (BBB) (32). Their depletion may exacerbate BBB disruption, as observed in DAI patients with elevated cerebrospinal fluid/serum albumin ratios (17). This mechanism parallels findings in severe TBI cohorts, where LPC reductions correlate with poor outcomes (11). In DAI patients, LPC 22:3 sn-2 reductions correlated with elevated CSF/serum albumin ratios (*ρ* = −0.48, *p* = 0.01), indicating BBB compromise. This mirrors findings in severe TBI cohorts, where LPC levels <2.5 μM predicted 6-month mortality with 82% sensitivity (17, 33). Mechanistically, LPC depletion may exacerbate neuroinflammation by promoting microglial activation and pro-inflammatory cytokine release (e.g., IL-6, TNF-α) (34, 35).

The integration of metabolomic and clinical data reveals that impaired FAO and disrupted glycerophospholipid metabolism form interconnected pathological hubs in DAI. This synergy creates a vicious cycle wherein mitochondrial dysfunction increases reactive oxygen species (ROS) production, leading to oxidative damage of phospholipids and exacerbating membrane instability (17, 36). Concurrently, the depletion of LPCs—critical components of HDLs—compromises cholesterol transport across the BBB, depriving neurons of substrates essential for myelin repair (32, 37). This dual pathology of energy failure and membrane degradation likely underlies the disproportionate white matter atrophy and cognitive decline observed in DAI survivors (10). The neuroinflammatory milieu in DAI is further amplified by elevated proline and indolelactic acid, metabolites linked to microglial activation and kynurenine pathway upregulation (38, 39). These changes correlate with calcium-mediated excitotoxicity, a hallmark of secondary axonal injury (40). Similarly, carnitine supplementation (3 g/day) enhanced mitochondrial biogenesis and attenuated oxidative stress in a pilot study of TBI patients (41, 42). If validated in DAI-specific cohorts, these interventions could address the critical unmet need for targeted neuroprotection.

Our pathway model’s superior diagnostic performance (AUC = 0.927) compared to traditional clinical markers (AUC = 0.744) emphasizes the value of multi-target approaches. This aligns with emerging trends in TBI research, where combining biomarkers from distinct pathophysiological axes (e.g., metabolic, inflammatory) improves predictive accuracy (10).

The identification of carnitine C8:1 and LPC 22:3 sn-2 as top discriminative metabolites opens avenues for targeted interventions. Carnitine supplementation has shown neuroprotective effects in preclinical TBI models, restoring mitochondrial function and reducing oxidative stress (43). Similarly, LPC analogs or HDL mimetics could stabilize axolemmal integrity, as demonstrated in experimental spinal cord injury (44).

The prognostic utility of our metabolic pathway model (AUC = 0.912 for GOSE prediction) also supports its integration into clinical workflows. For instance, early stratification of high-risk DAI patients could guide personalized neuroprotective strategies, such as hypothermia or anti-inflammatory therapies (20).

### 4.1 Limitations

This study has several limitations. First, the single-center design and stringent exclusion criteria may limit generalizability. Second, the absence of cerebrospinal fluid metabolomics precludes direct correlation between systemic and central nervous system metabolic changes. Third, reliance on LC–MS alone may overlook metabolites detectable via complementary platforms (e.g., GC–MS or NMR) (45).

Future studies should validate these findings in multi-center cohorts, incorporate longitudinal sampling to track metabolic recovery, and explore circadian variations in metabolite levels (46). Mechanistic studies in animal models could further elucidate causal links between FAO impairment and axonal injury, while clinical trials might test interventions such as carnitine supplementation or LPC analogs.

## 5 Conclusion

Our findings propose that DAI is associated with coordinated disruptions in lipid and energy metabolism, which may contribute to its pathophysiology and clinical progression. The integration of metabolic pathway signatures with clinical parameters offers a plausible strategy to enhance diagnostic precision. These insights may ultimately advance personalized management approaches for patients with DAI, pending confirmation through translational and clinical research.

## Data Availability

The original contributions presented in the study are included in the article/Supplementary material, further inquiries can be directed to the corresponding author.
